# HyperSHArc: Single-Isocenter Stereotactic Radiosurgery of Multiple Brain Metastases Using Proton, Helium, and Carbon Ion Arc Therapy

**DOI:** 10.1016/j.adro.2025.101763

**Published:** 2025-03-17

**Authors:** Lennart Volz, Peilin Liu, Thomas Tessonnier, Xiaoda Cong, Marco Durante, Andrea Mairani, Wenbo Gu, Amir Abdollahi, Xuanfeng Ding, Christian Graeff, Taoran Li, Stewart Mein

**Affiliations:** a Biophysics, GSI Helmholtz Centre for Heavy Ion Research GmbH, Darmstadt, Germany; b Department of Radiation Oncology, Corewell Health, Royal Oak, Michigan; c Heidelberg Ion Beam Therapy Center (HIT), Heidelberg, Germany; d Clinical Cooperation Unit Radiation Oncology, German Cancer Research Center (DKFZ), Heidelberg, Germany; e Heidelberg Institute of Radiation Oncology (HIRO), German Cancer Research Center (DKFZ), Heidelberg, Germany; f German Cancer Consortium (DKTK), Heidelberg, Germany; g Department is Institute of Condensed Matter Physics, Institute of Condensed Matter Physics, TU Darmstadt, Darmstadt, Germany; h Division of Molecular and Translational Radiation Oncology, National Center for Tumor Diseases (NCT), Heidelberg University Hospital, Heidelberg, Germany; i National Centre of Oncological Hadrontherapy (CNAO), Medical Physics, Pavia, Italy; j Department of Radiation Oncology, University of Pennsylvania, Philadelphia, Pennsylvania; k Department of electrical engineering and information technology, TU Darmstadt, Darmstadt, Germany; l Department of Accelerator and Medical Physics, Institute for Quantum Medical Science, National Institutes for Quantum Science and Technology (QST), Chiba, Japan

## Abstract

**Purpose:**

This work presents a proof-of-concept study of HyperSHArc, spot-scanning hadron arc (SHArc) therapy for single-isocenter stereotactic radiosurgery of multiple brain metastases (MBMs). HyperSHArc plans using proton, helium, and carbon ions were compared with state-of-the-art volumetric modulated photon arc therapy.

**Methods and Materials:**

Treatment design and optimization procedures were devised using commercial and in-house treatment planning systems. Planning and delivery methods considered dedicated energy, spot, and multiarc selection strategies. Proton, helium, and carbon HyperSHArc plans were generated for patients with MBM exhibiting 3 to 11 intracranial lesions with gross tumor volumes (GTVs) between 0.03 and 19.8 cc, at prescribed doses between 19 and 21Gy in a single-fraction. Planning target volumes (PTVs) considered a 1-mm isotropic margin around the GTV, and robust optimization with 2.5%/1 mm criteria for range and position uncertainty was applied. Photon hyper-arc volumetric modulated arc therapy (HA-VMAT) plans were optimized for the PTVs using the HyperArc® single-isocenter stereotactic radiosurgery platform (Varian, Palo Alto, CA, USA).

**Results:**

HyperSHArc plans were comparable between particle species, achieving highly conformal target doses and satisfying clinical coverage criteria. Particle arc plans reduced V_2Gy_ and V_4Gy_ in the healthy brain compared with HA-VMAT, while intermediate doses (V_8Gy_-V_16Gy_) were similar or reduced depending on the number of lesions. Particularly for the case with 11 targets, a considerable reduction in V_12Gy_ was observed that could be relevant for reducing the risk of treatment-induced radionecrosis. HyperSHArc using carbon ions boosted dose-averaged linear energy transfer inside the target relevant to overcoming radioresistance factors (>100 keV/μm).

**Conclusions:**

We present the first particle arc therapy strategies for MBM. Results demonstrate that with HyperSHArc, dose conformity comparable or superior to HA-VMAT is achievable while reducing the low-dose bath and increasing mean dose-averaged linear energy transfer in the GTV. Our findings suggest that HyperSHArc using light and heavy ions could be an effective and efficient means of treating MBM. Further development of HyperSHArc optimization and delivery is justified.

## Introduction

Multiple brain metastases (MBM) are the most common type of brain lesion and are commonly treated with stereotactic radiosurgery (SRS) or whole-brain radiation therapy.^1^^,^^2^ The steep dose gradients achievable with SRS afford better sparing of healthy brain tissue, permitting higher doses to the target while preserving neurologic function. The standard of care involves, when available, the application of dedicated SRS systems, such as Gamma Knife and CyberKnife.^3^^,^^4^ A recent prospective randomized phase 3 trial reported the superiority of single-fraction (24 Gy) treatment schedules over a three-fraction (3 × 9 Gy) regimen, indicating lower local recurrence rates and suppression of distant metastases.^5^ For patients with ≳4 lesions, long treatment delivery times, as well as the excess dose given to the healthy brain in these cases, has prompted the development of single-isocenter LINAC-based SRS planning and delivery techniques, for example, hyper-arc volumetric modulated arc therapy (HA-VMAT) by Varian. HA-VMAT has proven to be more time effective because of the simultaneous treatment of multiple lesions while offering acceptable tumor control and toxicity levels.^6^ Despite these advances, treatment of MBMs can be challenging, limiting the applicability of HA-VMAT over the Gamma Knife or CyberKnife techniques, especially in patients with many lesions or tumors close to critical structures.

Particle therapy, for example, proton, helium ion or carbon ion therapy, is a high-precision radiation therapy modality which could be beneficial in the treatment of MBM; however, to date, clinical application of particle therapy in the brain is mostly limited to single moderately sized targets using lower to mid-range dose levels (<300 cGy/fx). Particle therapy uses the steep dose gradient of ion beams to deliver a highly conformal target dose while minimally affecting surrounding healthy tissue.^7^ Historically, the Bragg peak characteristic of ion beams was proposed as an appropriate tool for stereotactic radiosurgery^8^ and has proven to be effective in treating several sites, including extracranial metastases, for example, in the liver.^9^ A recent work presents a retrospective analysis to investigate proton therapy in the context of MBM treatment compared with photon therapy.^10^ The study was based on passive-scattering proton beam delivery with custom beam shaping devices (eg, collimators), which is not common practice in modern-day pencil-beam scanning proton therapy machines. Still, promising outcomes were reported that warrant further research in improving SRS delivery with particle therapy.

In order to apply SRS techniques using particle therapy, several key challenges must be addressed. Uncertainties affecting the physical and biological dose distribution, because of uncertainties in the particle range, dose-averaged linear energy transfer (LET_d_), and lateral scattering, have to be accounted for and need to be minimized to attain the level of precision needed to treat small lesions of a few millimeters in diameter using SRS techniques.^11^ High fraction doses are also a challenge for contemporary models that predict the relative biological effectiveness (RBE).^12^ Dynamic beam collimation or using ions heavier than protons could reduce the lateral beam penumbra and, therefore, the integral dose to the brain. However, given the current state of pencil-beam scanning delivery with a few static beam angles, treatment of MBM could be particularly challenging and time-consuming for routine clinical application.

The development of novel treatment delivery strategies in particle therapy could improve dose conformity and delivery efficiency as required for SRS. For instance, particle arc therapy (ARC) techniques, such as spot-scanning proton arc (SPArc) and spot-scanning hadron arc (SHArc) therapy, have been developed as a method to increase dose conformity and mitigate uncertainties.^13^^,^^14^, ^15^, ^16^ Instead of applying the treatment through a few fields, as performed currently in particle therapy, the ARC concept implies particle beam delivery using one or multiple arcs around the patient, similar to VMAT treatments. With advances in planning strategies and delivery techniques, dynamic delivery of proton arc therapy using a gantry system has been demonstrated to be more time efficient compared to approaches with few fields.^17^^,^^18^ Treatment planning studies indicate that SHArc delivery times are similar to multifield heavy ion plans.^14^, ^19^ The increased degrees of freedom and flexibility in placing the Bragg peak enabled by ARC permits unprecedented dose shaping and also enables LET_d_ optimization, focusing the high LET_d_ inside the target, while reducing it in the healthy tissue.^14^^,^^20^ This is particularly the case for heavy ions, where the LET_d_ focusing can reach levels of >100 keV/µm, relevant to overcoming radioresistance factors, for example, induced by tumor hypoxia.^14^, ^19^, ^21^ A recent study has already shown the potential of proton arc therapy for treating single metastases.^22^ The dosimetric benefits seen with ARC for various cancer types are expected to translate also to multimetastases SRS.^11^ However, no study has yet explored the use of proton or ion ARC for MBM treatment in comparison to the state-of-the-art HA-VMAT photon-based SRS.

**Figure 1:**
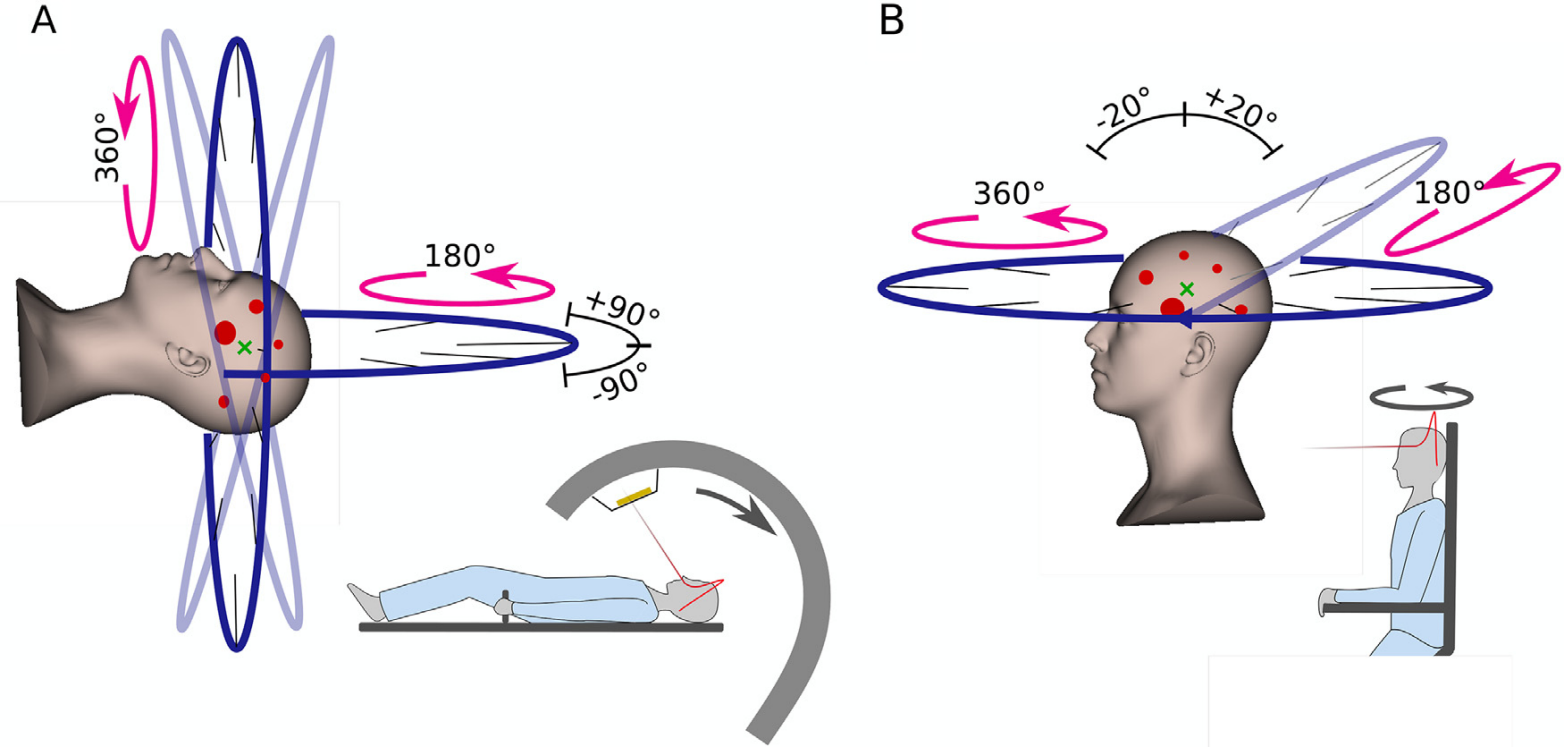
Schematic of HyperSHArc setup and delivery for (A) gantry-based and (B) upright particle therapy. Gantry and couch kick together offer the option of choosing various noncoplanar subarcs. Similar flexibility can be achieved with an upright positioned patient rotated in front of a fixed horizontal beamline.^23^

Through a multi-institutional collaboration, planning and optimization strategies were developed for treating MBM using SRS proton, helium, and carbon ion arc (HyperSHArc) therapy. The HyperSHArc frameworks facilitate both iterative and heuristic energy selection procedures, selection of subarcs, as well as noncoplanar trajectories (Fig. 1).^23^ In this work, HyperSHArc is demonstrated as a proof-of-concept study for 3 different patient cases exhibiting 3, 5, and 11 lesions. HyperSHArc plans were compared with state-of-the-art HA-VMAT in terms of potential dosimetric benefit, achievable plan quality, and delivery duration.

## Methods and Materials

### Patient cohort

Patient data were procured from the publicly available Varian Medical Affairs online database (https://medicalaffairs.varian.com/edge-case-studies). Three patients with 3 (P3m), 5 (P5m), and 11 (P11m) brain lesions, respectively, were selected. The case with 11 lesions included a larger resection cavity. The patient specifics are reported in Table 1.

**Table 1:** Characteristics of the investigated multiple brain metastases patient cases

Patient	Number of targets	Median (min–max) target volume [cc]	Prescribed dose
P3m	3	0.31 (0.28-0.41)	21 Gy (All)
P5m	5	3.39 (0.5-4.14)	21 Gy (All)
P11m	11	0.1 (0.03-19.9)	21 Gy (Lesions) 19 Gy (Resection cavity)

### Treatment planning

#### HyperSHArc

Treatment delivery efficiency is directly linked to the energy selection strategy underlying the HyperSHArc plan optimization. Energy switching times cause delays in the overall treatment duration, and therefore, minimizing energy layer switching time by reducing the overall number of energy layers and choosing the optimal sequence of energy layers has been a key topic of research in the ARC community.^13^^,^^24^^,^^25^ Energy layer switching is particularly a concern for heavy ion beams, where every energy change is typically associated with a new acceleration cycle and a spill pause of a few seconds duration^19^.

The HyperSHArc using protons (HyperSHArc-p) optimization algorithm is based on the iterative SPArc optimization algorithm^26^ through an in-house developed Python script and implemented in RayStation (RaySearch, Stockholm, Sweden). Treatment plans consisted of 3 noncoplanar partial arcs with 5° sampling frequency, leveraging the possibilities of gantry-based arc delivery. Energy selection is then done through iterative optimization and energy layer redistribution through the Python script.

HyperSHArc using carbon ions (HyperSHArc-C) optimization algorithms were developed and implemented in TRiP98.^27^ For heavy ions, the variable RBE further increases optimization complexity.^14^, ^19^ For calculation and optimization of effective dose, HyperSHArc-C applied the local effect model version IV (LEM-IV) RBE model^28^ with a global α/β ratio of 2Gy. To enable robust optimization for HyperSHArc-C in TRiP98, several steps for increased efficiency were implemented, for example, a more efficient setup of angular control points, and improved memory efficiency of the optimization algorithm. For focusing the high LETd inside the target, the monoenergetic ARC optimization approach presented by Bertolet and Carabe^25^ was applied. To compute the central energy, we first used a distance transform of the target voxels to identify the target boundary and performed a ray-tracing algorithm to infer the water equivalent depth only on the boundary voxels. This then permits a fast way to determine the central water depth as needed for the approach by Bertolet and Carabe. To further streamline the arc control point setup, a “template” control point was created, of which each additional control point was simply a copy with just few key variables (field direction, energy) exchanged. With gantries for heavy ion beams being a rare commodity, HyperSHArc-C followed an envisioned delivery strategy based on a fixed beamline and upright positioned patient,^23^^,^^29^ such as already available at the Shanghai Proton and Heavy Ion Center.^30^ HyperSHArc-C plans considered full coplanar arcs for each metastasis, comprising 120 control points each, separated at a 3 degree angular step. The center of mass of the gross tumor volumes (GTVs) was set as the treatment isocenter, except in the case of patient P11m, where a shift was introduced to accommodate the distribution of metastases and the limited available field size of 20 × 20 cm^2^ at clinical carbon facilities.

In all HyerSHArc plans, planning target volumes (PTVs) extended GTVs by an isotropic 1 mm margin, and robust optimization assuming 1 mm/2.5% position/range uncertainty was applied. These criteria can be assumed conservative given dual-energy computed tomography availability for treatment planning.^31^ Constraints were chosen to meet D99% ≥ 100% in the GTV. Target overdose was not considered in the optimization, as a means to enable the steepest possible dose gradients, as recently established for photon VMAT SRS.^32^ The plans were optimized, and dose calculation was performed on a subsampled grid of 1 mm^3^ voxels. In the case of HyperSHArc-C plans, avoidance rings around the target were added to the optimization with a low weight to improve conformity, while the patient body was not considered in general, for computational efficiency.

#### Hyper-arc VMAT

Hyper-arc VMAT plans were prepared with the Eclipse (Varian, Palo Alto, CA, USA) treatment planning software using the Varian HyperArc solution with the 10XFFF photon energy setting. PTVs extended the GTVs by an isotropic 1 mm margin. As for HyperSHArc, the optimization prioritized target coverage, that is, D99% ≥ 100%, without consideration for overdose.

### Evaluation

Photon plans were evaluated in terms of physical dose (Gy), whereas particle plans were evaluated in terms of RBE-weighted doses (GyRBE). For simplicity, we use a common dose scale in all presented figures and “Gy” for both Gy and GyRBE. Dose-volume histograms were computed for the target volumes, as well as the brain excluding the GTV. All plans were evaluated for the target coverage (D99%). Although target overdose and dose heterogeneity were not considered key quantities of interest for the comparison, they are provided for completeness. Dose conformity and homogeneity were assessed based on the conformity index and homogeneity index as per the Radiation Therapy Oncology Group definitions^33^:

$$CI=\;\frac{V_{RI}}{TV}$$

$$HI=\frac{I_{max}}{RI}$$

Where V_RI_ is the volume receiving the planned target dose, and TV is the total tumor volume. RI is the planned target dose, and I_max_ is the maximum dose in the target.

A key factor influencing the patient outcome is radiation-induced necrosis of healthy brain tissue. Previous studies suggested V_12Gy_ in the brain as a predictive factor for brain necrosis^34^^,^^35^; hence, this metric was chosen as the main point for comparison of healthy tissue doses in this work. In addition, further dose points were evaluated, such as V_2Gy_ and V_4Gy_ as metrics for the low-dose bath in the healthy brain.

### Delivery time estimation

HA-VMAT plans were delivered on a Varian TrueBeam® Edge with a high-definition 120 multileaf collimator (HD 120 MLC). At our institution, an mega-voltage image is acquired between each delivered subarc (after couch kicks for the subsequent arc) and checked against the simulated digitally reconstructed radiograph for mid-treatment positioning verification, alongside alignRT surface tracking. The time for imaging is not considered in the reported delivery times. It would result in an additional ∼90 seconds that can be added to the delivery times (ie, ∼30 seconds for each pause, including imager extension, retraction, acquisition, and human evaluation) if no out-of-tolerance motion is observed.

For HyperSHArc-p, the delivery time was simulated based on Liu et al^36^, which was benchmarked for a cyclotron accelerator. For HyperSHArc-C, delivery was simulated using a dedicated synchrotron simulation platform,^19^ assuming the beam parameters of the Heidelberg Ion Beam Therapy Center, where carbon and helium ion beams are used clinically. This includes realistic minimum gating duration of 0.3 second, accelerator cycles of 4 seconds spills with 4 second spill pause in between, and patient rotation with an upright positioning system at 1 rpm. Although a full validation against beam delivery for HyperSHArc-C remains outstanding, considering that no fully integrated SHArc-C delivery platform yet exists, the simulator uses known performance to approximate the true delivery. Layer delivery times were calculated based on the clinical beam model of the Heidelberg Ion Beam Therapy Center including dynamic intensity control for obtaining the best particle rate per spot. Patient rotation was realistic to medical device regulations (IEC 60609-1). Adjacent beam angles with the same energy were assumed to be deliverable within the same spill, with a 0.3 second gate in between for patient rotation. For adjacent beam angles with different energies, 2 approaches were investigated: First, multienergy extraction^37^ was assumed, where the beam energy changes in the time needed to rotate the patient between adjacent control points (0.3 second for 2 degree steps) based on the multienergy extraction modes at HIMAC^38^ and the Heidelberg Ion Beam Therapy Center.^37^ Second, plan delivery times were computed assuming a conventional energy switch through a full synchrotron acceleration cycle (4 seconds pause between energies).

## Results

### Dosimetric comparison

**Figure 2:**
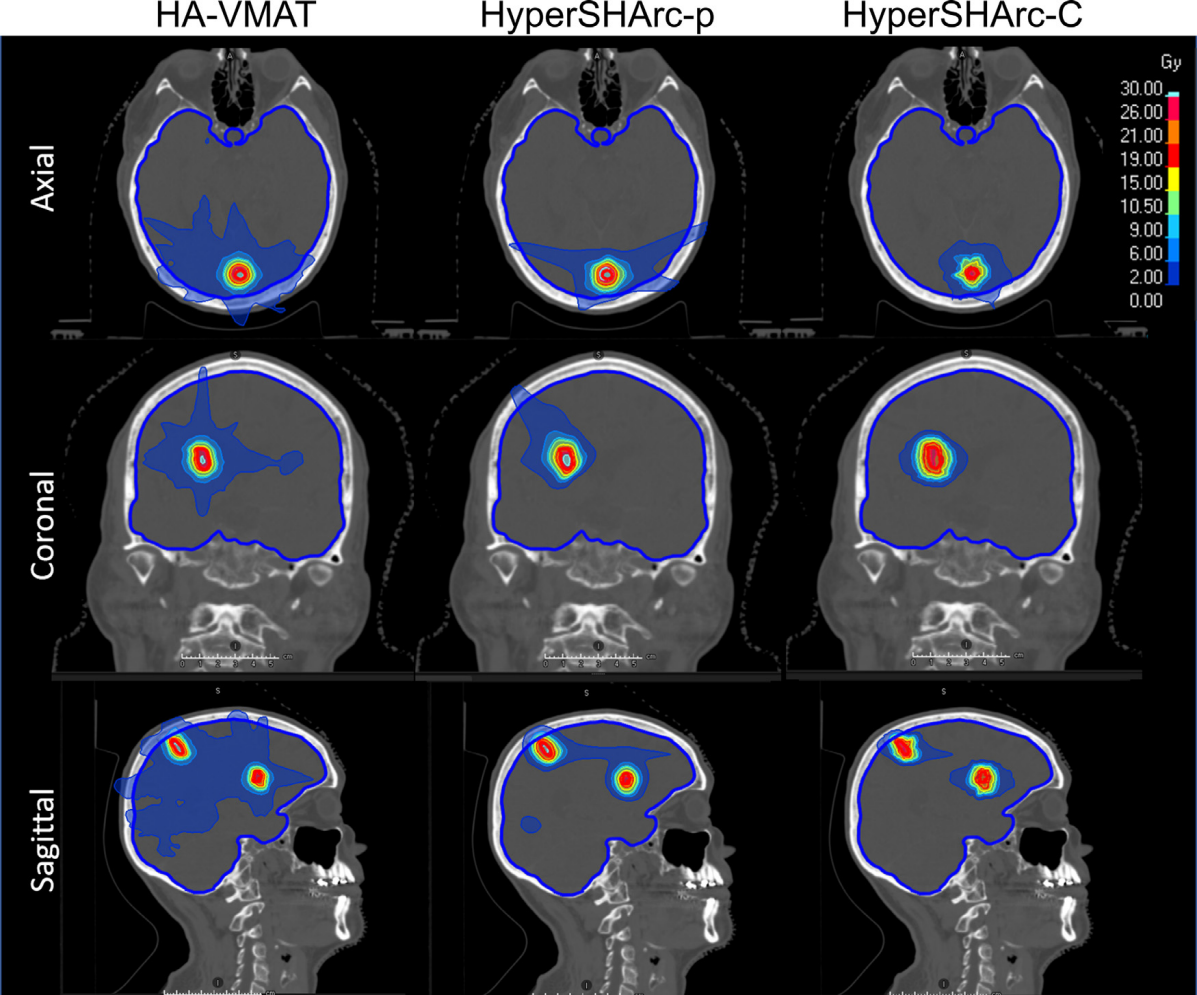
Dose maps for HA-VMAT (left), HyperSHArc-p (middle), and HyperSHArc-C (right) plan for P3m, using a 2Gy low-dose threshold. The HA-VMAT plans are presented in Gy, whereas the HyperSHArc plans are presented in GyRBE. *Abbreviations:* HyperSHArc-C = HyperSHArc using carbon ions; HyperSHArc-p = HyperSHArc using protons.

**Figure 3:**
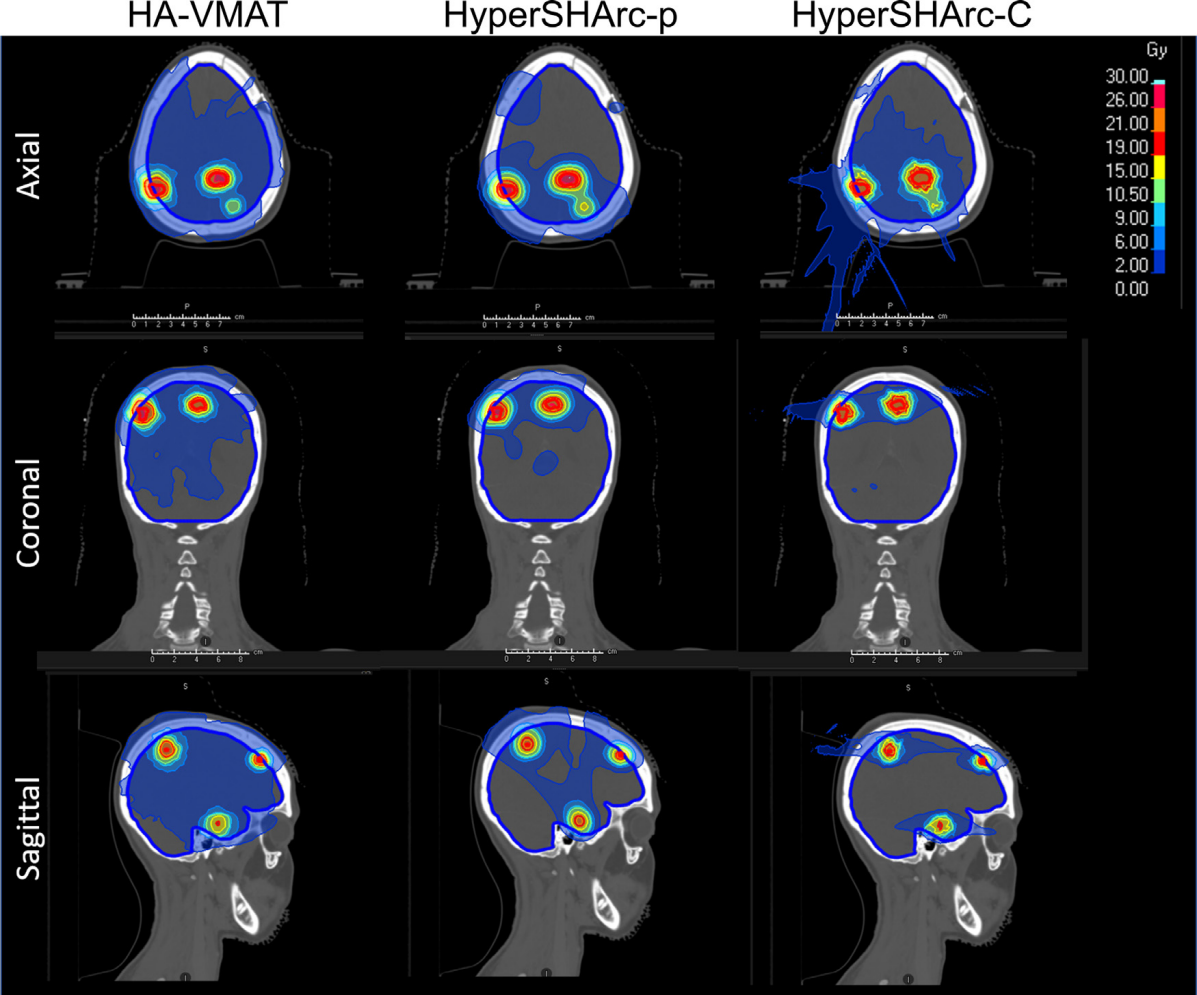
Dose maps for HA-VMAT (left), HyperSHArc-p (middle), and HyperSHArc-C (right) plan for P5m, using a 2Gy low-dose threshold. The HA-VMAT plans are presented in Gy, whereas the HyperSHArc plans are presented in GyRBE. *Abbreviations:* HyperSHArc-C = HyperSHArc using carbon ions; HyperSHArc-p = HyperSHArc using protons.

**Figure 4:**
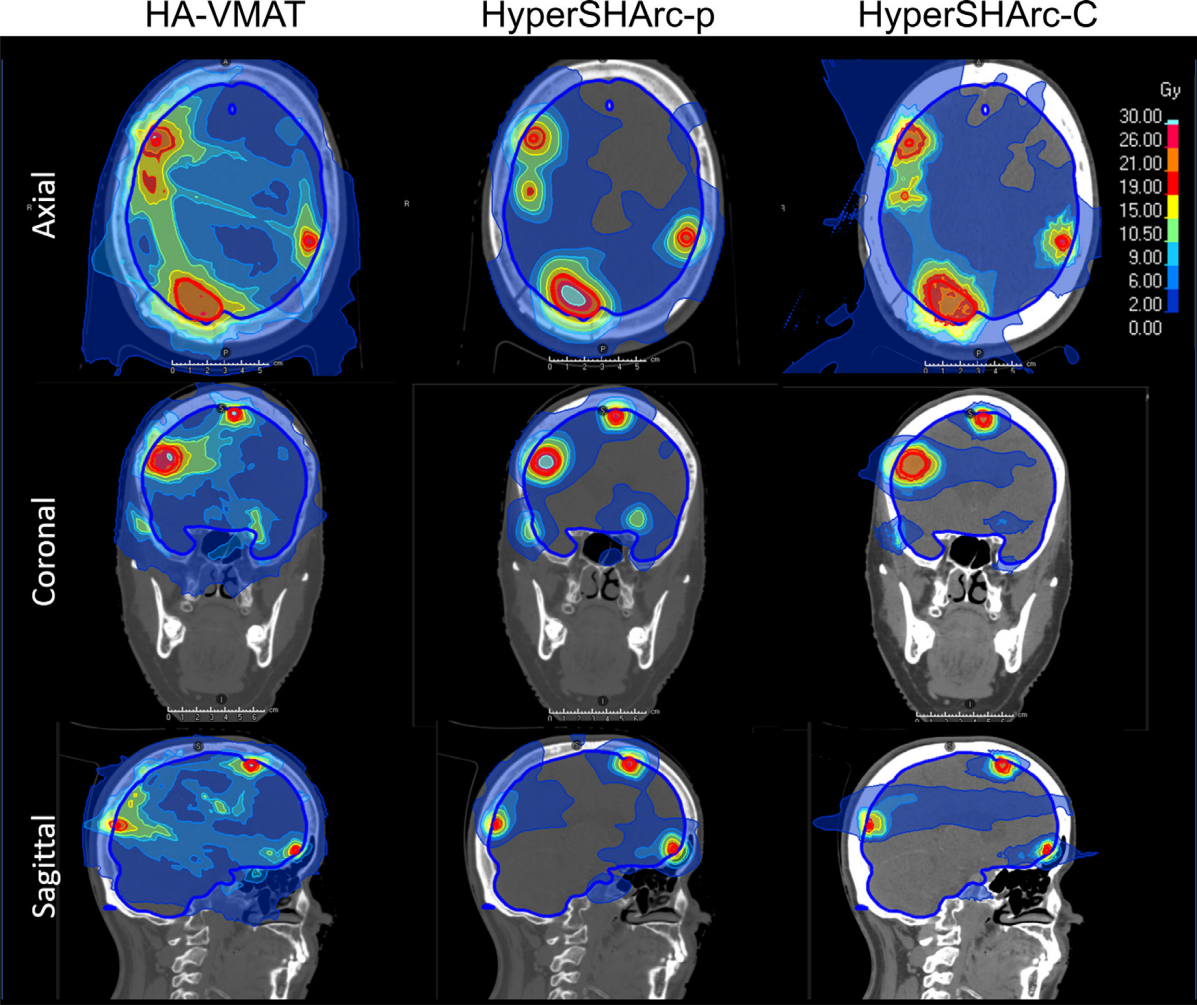
Dose maps for HA-VMAT (left), HyperSHArc-p (middle), and HyperSHArc-C (right) plan for P11m, using a 2Gy low-dose threshold. The HA-VMAT plans are presented in Gy, whereas the HyperSHArc plans are presented in GyRBE. *Abbreviations:* HyperSHArc-C = HyperSHArc using carbon ions; HyperSHArc-p = HyperSHArc using protons.

Figure 2 shows dose comparisons between the different modalities for 3 (P3m), Fig. 3 for 5 (P5m), and Fig. 4 for 11 metastases (P11m). HA-VMAT and HyperSHArc plans were highly conformal to the GTV. HyperSHArc plans show reduced low-dose bath compared to HA-VMAT, with further selective organ-at-risk dose-sparing potential when noncoplanar arc trajectories are applied.

**Figure 5:**
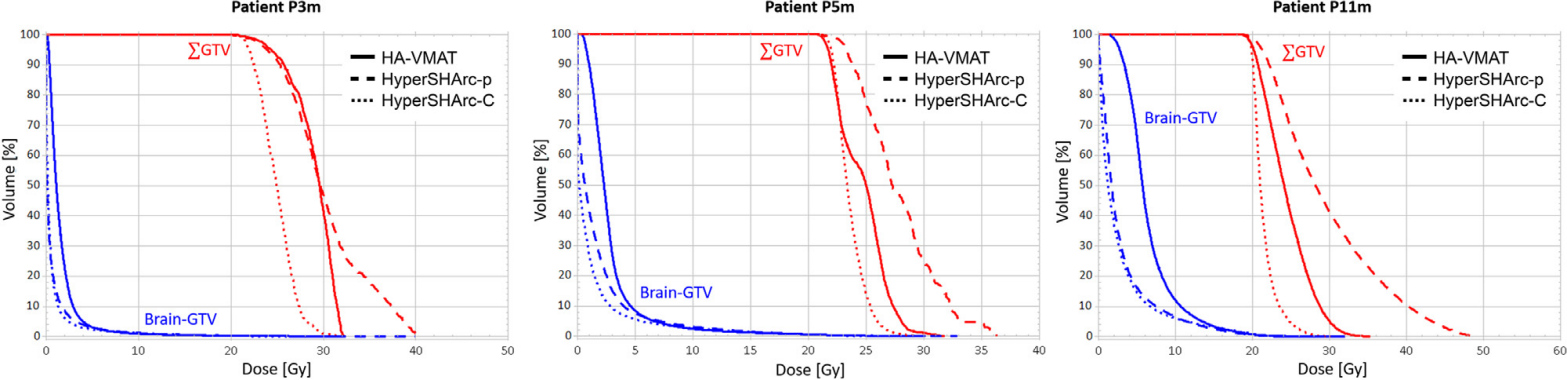
Comparison of the dose-volume histograms for 3 patients and different photon and particle stereotactic radiosurgery techniques. Left: P3m. Middle: P5m. Right: P11m. HA-VMAT (solid), the HyperSHArc-p (dashed), and HyperSHArc-C (dotted). The red curves show the combined targets, whereas the blue curves show the healthy brain. For P11m, the dose of one of the 11 GTVs (the resection cavity) was 19 Gy, and the rest of the GTVs were planned to be 21 Gy. For the HyperSHArc plans, the low-dose bath to the healthy brain was considerably reduced at a similar lower V12Gy compared to HA-VMAT plans. The HA-VMAT plans are presented in Gy, whereas the HyperSHArc plans are presented in GyRBE. *Abbreviations:* HyperSHArc = Hyper spot-scanning hadron arc; HyperSHArc-C = HyperSHArc using carbon ions; HyperSHArc-p = HyperSHArc using protons.

The dose-volume histograms for the 3 patients for each modality are provided in Fig. 5, and the detailed dosimetric results are listed in Table 2. HyperSHArc-p plans showed an increased overdose compared with HA-VMAT and HyperSHArc-C, which was a consequence of the fact that overdose was not considered in the plan optimization. HyperSHArc-C plans were more homogeneous and conformal to the target volume than HyperSHArc-p plans, with similar homogeneity index and conformity index compared with HA-VMAT. V_12Gy_ in the healthy brain was slightly elevated for particle arc plans for P3m and P5m compared to HA-VMAT, being larger by 2.9/2.3 cc and 8/4.4 cc, respectively, for HyperSHArc-p/HyperSHArc-C. The appreciable advantage for the particle arcs was observed for P11m, where HyperSHArc-p and HyperSHArc-C plans reduced V_12Gy_ by 34.7 and 32.7 cc, respectively. HyperSHArc-p and HyperSHArc-C showed a greatly reduced low-dose bath (V_2Gy_ and V_4Gy_) in the healthy brain compared with the HA-VMAT plans, with the difference in V_2Gy_ becoming as large as 790 cc in the case of patient P11m. Compared with the planned dose, D1% was increased by an average of 51%, 69%, and 40% for P3m, 33%, 45%, and 28% for P5m, and 35%, 41%, and 34% for P11m for HA-VMAT, HyperSHArc-p, and HyperSHArc-C, respectively.

**Table 2:** Dose-volume histograms metrics for 3 patients and stereotactic radiosurgery plans are generated for different modalities. For the GTVs, averaged values are provided for better comprehensiveness. The metrics central to the discussion in this work are highlighted in light blue. The HA-VMAT plans were evaluated in physical dose, whereas the HyperSHArc plans were evaluated in relative biological effectiveness-weighted dose

Patient	Modality	GTVs (averaged)				Healthy brain [cc]					
		D99%[Gy]	D1% [Gy]	Homogeneity index	Conformity index	V2Gy	V4Gy	V8Gy	V10Gy	V12Gy	V16Gy
**P3m**	HA-VMAT	25.4	31.8	1.5	3.8	342.4	71.7	18.8	12.3	8.8	5.1
	HyperSHArc-p	24.9	35.4	1.7	4.3	123.5	55.4	22.5	15.9	11.7	6.6
	HyperSHArc-C	21.3	29.4	1.4	2.5	88.9	42.4	20.3	15.0	11.1	5.7
	Carbon IMPT	21.1	22.8	1.1	2.2	111.3	48.5	20.3	15.7	12.1	6.6
**P5m**	HA-VMAT	24.6	28.0	1.3	4.5	776.0	171.4	45.2	29.9	21.5	12.4
	HyperSHArc-p	22.1	30.5	1.5	3.0	330.7	127.5	53.8	39.7	29.5	15.3
	HyperSHArc-C	21.5	26.9	1.3	2.7	189.5	88.0	43.5	33.6	25.9	14.3
	Carbon IMPT	21.1	22.8	1.1	1.5	176.0	102.4	40.5	30.3	23.2	13.1
**P11m**	HA-VMAT	22.6	28.1	1.4	4.1	1278	1067	279.5	157.9	92.9	33.2
	HyperSHArc-p	21.9	29.0	1.4	3.7	564.9	271.3	118.2	83.4	58.2	25.1
	HyperSHArc-C	21.3	27.8	1.4	3.7	488.0	250.5	102.9	78.2	60.2	33.0
	Carbon IMPT	21.0	22.2	1.1	6.8	392.8	222.3	138.5	87.2	72.5	43.4

*Abbreviations:* HyperSHArc = Hyper spot-scanning hadron arc; HyperSHArc-C = HyperSHArc using carbon ions; HyperSHArc-p = HyperSHArc using protons.

### Comparison to IMPT

As a point of reference, we compared the HyperSHArc-C plans to a carbon intensity-modulated particle therapy (IMPT) approach with 3 fields (2 lateral and 1 vertex field). The results were added to Table 2. The plans all met the D99% > 100% criteria set out as a target for the optimization. For P3m and P5m, the IMPT plan produced a similar dose to the healthy brain compared to HyperSHArc-C. For P11m, although carbon IMPT yielded a reduced low-dose bath, this came at the expense of an increase in the intermediate doses, notably also in V12Gy which was 12 cc higher than that of HyperSHArc-C. In addition, for P11m, the carbon IMPT plan was not very conformal to the target volumes. An example dose slice for P11m is provided in the supplementary material.

### Delivery time

HA-VMAT plans were delivered on a Varian TrueBeam Edge with a high-definition 120 multileaf collimator (HD 120 MLC). From start to end of irradiation, the HA-VMAT plans, which comprised 4 arcs with 3 corresponding couch kicks, took 7 minutes and 20 seconds for P3m, 7 minutes and 32 seconds for P5m, and 6 minutes and 45 seconds for P11m. For patient treatment, mega-voltage images are often acquired between each delivered arc and are performed and compared to digitally reconstructed radiographs from the planning CT to ensure patient positioning accuracy, alongside surface guidance. The position verification process normally takes ∼30 seconds, such that ∼90 seconds may be added to the above delivery times.

In comparison, HyperArc-p plans, which comprised 3 arcs each, took 3 minutes 43 seconds for P3m, 8 minutes and 40 seconds for P5m, and 7 minutes and 1 seconds for P11m. This time is subject to the scanning speed for the spot-scanned delivery, the gantry rotation speed, and the energy layer switching time, as determined by the beam delivery simulator in Liu et al.^36^ For HyperSHArc-C, plan delivery as determined by our in-house beam delivery simulator^19^ took approximately 8 minutes for P3m, 16 minutes for P5m, and 35 minutes for P11m, when multienergy extraction was assumed. The duration was approximately tripled when a regular synchrotron acceleration cycle for energy switching was enforced. Note that both assume upward and downward shifts to be of the same time duration, which may not be feasible, because of magnet hysteresis.

For HyperSHArc plans, additional imaging may be necessary, depending on the protocol, similar to the HA-VMAT plans. Because no clinical protocol yet exists for these plans, additional time for imaging was not estimated here.

## Discussion

HyperSHArc marks the first ARC technique for treating MBM using protons and other ions. This was achieved by developing infrastructure in both clinically used and research treatment planning systems, building on the latest advances available to the ARC community, such as efficient energy selection strategies. The proof-of-concept for HyperSHArc SRS was drawn using publicly available patient data procured from the Varian Medical Affairs online database (Varian). Results for HyperSHArc planning and optimization were compared against state-of-the-art HA-VMAT plans for the patients. Overall, promising dosimetric quality was achieved, with HyperSHArc providing similar or better healthy brain dose parameters compared with HA-VMAT SRS.

### Dosimetric comparison of HyperSHArc and other treatment techniques

HyperSHArc and HA-VMAT plans were designed to meet D99% > 100% criteria on the GTV while reaching a steep dose gradient outside the PTV. To accommodate this, overdose in the target was tolerated and not included in the optimization criteria. Overall, HyperSHArc-p yielded the most overdose to the target probably a result from the robust optimization. Despite not being weighted into the objective function, carbon IMPT and HyperSHArc-C delivered the most homogeneous plans, because of the precision offered by the ^12^C ion Bragg peak. Interestingly, a recent clinical study^32^ has demonstrated significantly improved tumor control for inhomogeneous dose distributions compared with a homogeneous dose prescription (1-year local control rate of 93% vs 78%). In the study, homogeneous target dose prescription was compared with inhomogeneous dose prescription, where the PTV contour was constrained to a similar dose level as for the homogeneous plans, while the isocenter was set to receive ∼40% increase in dose. This finding set the basis for not including overdose as an objective, although clinical protocols may still require more homogeneous dose distributions compared to those presented in this work. In that case, additional parameter optimization specific for overdose would be needed, which was outside the scope of this work.

In this work, we focused on V_12Gy_ as a main point of comparison for healthy tissue doses. Daisne et al^39^ retrospectively analyzed patients treated for 1 to 3 lesions per fraction and identified V_12Gy_ as a main risk factor for radionecrosis. They showed the probability for radionecrosis to increase monotonously with increasing V_12Gy_, such that a reduction of this parameter is paramount for reducing treatment toxicity. A recent work^10^ has reported on a retrospective clinical study involving 370 patients using passively scattered proton beams to treat patients with MBMs. In their work, proton beam therapy proved an effective and safe treatment for MBM, where for cases with 3 and 4 lesions, similar V_12Gy_ levels were observed between proton therapy and LINAC-based photon therapy. Still, 26 patients developed radionecrosis, predominantly those with reirradiation, with the median time to develop radionecrosis reported comparable to photon SRS. Their analysis further showed a correlation of radionecrosis with tumor volume, in-line with higher intermediate healthy brain dose volumes.

Despite the differences in overdose levels to the target, all plans showed comparable intermediate doses to the healthy brain. For P3m and P5m, HA-VMAT, carbon IMPT, and HyperSHArc plans yielded similar V_12Gy_ in the healthy brain. For P11m, HyperSHArc plans considerably reduced V_12Gy_ compared with HA-VMAT, by 33 and 35 cc for HyperSHArc-p and HyperSHArc-C, respectively, and even up to 12 cc compared with carbon IMPT. This indicates promising potential for future application of HyperSHArc for patients with a larger number of metastases (>5), which are in general the most challenging to treat. For all cases, HyperSHArc plans produced a sizable reduction in the low-dose bath compared with HA-VMAT plans, more than halving V_2Gy_ for P11m. This feature of HyperArc plans is a direct result of the favorable dose and, in the case of heavier ions, RBE distributions of the ion beams. As an intermediate step between protons and carbon ions, we also investigated HyperSHArc using helium ions (see Supplementary material). For HyperSHArc-He, further potential was observed for P5m (Fig. E2) with reduced dose to the healthy brain compared to HyperSHArc-p and HyperSHArc-C plans and better target homogeneity compared to HyperSHArc-p. Considering the technologically simpler acceleration of helium compared with carbon ions, HyperSHArc-He could be an interesting middle ground between HyperSHArc-p and HyperSHArc-C, as is the case also in IMPT.^40^^,^^41^ It is important to consider that all ion plans included robust optimization on top of the 1mm isotropic PTV margin applied for HA-VMAT. This follows current clinical protocols in both photon and ion treatments. The best robustness approach for HyperSHArc, however, remains to be decided based on the uncertainties of ARC delivery systems.

The increased degrees of freedom in optimization and delivery using HyperSHArc afford the ability to boost the LET_d_ inside the target lesions. In the case of HyperSHArc-C, LET_d_ in the target reached values beyond 120 keV/μm for all 3 patient cases (see Supplementary material). This feature of HyperSHArc may lead to improved lesion targeting because tumor radioresistance is known to be overcome by increased LET_d_,^42^ which could be particularly helpful for reirradiation of recurrent tumors. The small target volumes for MBM treatments ensure that most of the target dose in HyperSHArc therapy is delivered by the Bragg peak, which, for carbon ions, also corresponds well to the point of highest LET_d_.^43^ This makes carbon ions, and particularly HyperSHArc-C, a prime candidate to increase LET_d_ to small lesions.

### Application of RBE models for HyperSHArc

It is important to note that in this study of HyperSHArc, the direct application of existing RBE models is an additional source of uncertainty because of the increased differences in model predictions for extreme hypofractionation, that is, dose levels ≥10GyRBE per fraction.^12^^,^^44^ For HyperSHArc-C, we used a state-of-the-art RBE model, LEM IV, to estimate the RBE-weighted dose. Clinically, all European carbon ion therapy centers currently still employ LEM I,^45^ despite evidence that other models may provide more accurate estimates of the dose, LET, and tissue type dependencies.^46^ Compared with the newer LEM IV version used here, a LEM I-based optimization for patients with MBM resulted in increased healthy tissue doses, but it is known that LEM I does not present the correct RBE-weighted dose. Particularly, it underestimates the RBE at high LET_d_, hence causing higher entrance doses for HyperSHArc-C. Recent works comparing LEM IV and mMKM against in vivo RBE measurements show an acceptable agreement for high-dose-per-fraction irradiation of the rat spinal cord between 20 and 30 Gy.^46^^,^^47^ Although LEM IV may be a more reasonable choice compared with LEM I because of the high LET_d_ focus in the lesions and the assumed single-fraction delivery, we expect larger uncertainties in the RBE-weighted doses for HyperSHArc-C. Similarly, the validity of constant RBE of 1.1 is likely not accurate for HyperSHArc-p in this work because the conservative estimate is based on experimental findings for mid-spread-out-Bragg-peak irradiations at standard fraction doses,^12^ typically ∼2 Gy/fx.

Nonetheless, the results presented here are promising and provide a basis to justify further development of HyperSHArc for MBM treatment. Further studies are warranted to investigate the most appropriate definition of RBE for HyperSHArc therapy, for example, simulation studies, preclinical experiments, and advanced RBE modeling.

### Delivery feasibility

The HyperSHArc-p plans were competitive in delivery efficiency compared with the HA-VMAT deliveries. For P3m, even a slight benefit was observed for HyperSHArc-p compared with the HA-VMAT plan at a 3.5 minutes shorter delivery time. For HyperSHArc-C, plan delivery as determined by our in-house beam delivery simulator took considerably longer, being competitive only for P3m; the delivery times were nearly linearly increasing with the number of treated lesions because we assumed a single, slow patient rotation per lesion. This was done because of the employed energy selection strategy, which used a single energy layer per metastasis, aimed at the lesion's mean water equivalent depth. Because of the lesions’ small sizes and different treatment depths in the patient, it is not always possible to find an energy that can target multiple lesions at the same time at a given control point. Recent algorithms, such as energy layer preselection and filtering,^24^^,^^48^ may enable to obtain similar or better quality compared to the HyperSHArc-C plans in this work at more efficient delivery times if they can be ported to HyperSHArc-C planning. This is challenging because of the carbon ions variable RBE and the resulting non-convex optimization problem with increased computational demand, but will be investigated in future work.

For HyperSHArc-C delivery, we assumed the availability of multienergy extraction as discussed in Mein et al^15^, ^16^, which is applied clinically at QST^49^ and is currently under development at the Heidelberg Ion Beam Therapy Center.^37^ This mode permits reacceleration (or deceleration) of the beam in the synchrotron, which enables fast (in the order of 100 ms) energy layer switching times. Hence, we assumed the energy layer switching to be completed within the time needed to rotate the patient between adjacent control points. However, the final delivery energy sequence and the resulting delivery speed will depend on the exact implementation of the multienergy extraction mode and are subject to further investigations. With the current clinical beam delivery parameters at the Heidelberg Ion Beam Therapy Center, where all beam energy changes require a full acceleration cycle, treatment delivery was a factor of 3 longer for the HyperSHArc-C plans. Note that for the reported times, we also assumed that upward and downward energy shifts are equal in duration, which is likely not the case, because of magnet hysteresis. In the case of substantial differences between the two energy change directions, reordering of the arc delivery could be feasible, using that the patient or gantry can be rotated much faster than the total beam delivery, to minimize direction changes in the energy switching at delivery time.

Gantry-based delivery was assumed for HA-VMAT and HyperSHArc-p plans, which permitted to plan for multiple noncoplanar arcs to achieve the best result. Currently, there are 110 proton therapy centers worldwide treating with gantries, that is, almost all of the 119 total proton therapy facilities.^50^ For carbon ions, of the 14 facilities in operation, only 4 currently offer gantry-based treatment, and for those sometimes, only a limited number of beam angles are commissioned.^14^ Hence, realizing HyperSHArc-C will likely involve a fixed-beam nozzle and upright patient position system, rotating the patient instead of the beam. However, this comes with its own challenges, such as anatomic differences between the upright and supine positions.^29^ Patient motion during the slow rotation needs to be monitored to ensure accurate dose delivery, particularly in the case of SRS, where the highest precision is required, and additional verification imaging may be needed. However, for intracranial targets, such as MBM, no relevant anatomic differences between supine and upright are expected,^29^ and the change in posture does not require any differences in treatment planning approach except a simple coordinate transform. Nevertheless, studies are needed that explore the dosimetric uncertainty associated with slow patient rotation in an upright position.

### Future implementation in clinical treatment planning systems

HyperSHArc-p plans were generated using an external extension script added to RayStation using the SPArc algorithm.^13^ The presented results, therefore, are representative of what could be translated to clinical application as of now because plans were also deliverable within clinically reasonable time. As a future advanced option, this article highlights HyperSHArc-C, which provides further dosimetric and biological advantages. As outlined earlier, this strategy still requires improved delivery strategies, but also an implementation into clinical treatment planning systems. For this work, HyperSHArc-C plans were built on top of the ion ARC platform in TRiP98^19^ because TRiP98 is flexible, well-validated experimentally for various applications,^51^ and offers the potential for memory efficiency and runtime optimization. SHArc and HyperSHArc planning strategies in commercial systems are under continuous development, with recent works showing several interesting approaches.^14^, ^15^, ^16^, ^21^

## Conclusion

We present the first study for treating MBM using SHArc. Achieving target dose conformity comparable to or better than state-of-the-art HA-VMAT at improved low-dose bath, HyperSHArc using protons or carbon ions could be a promising candidate for MBM treatments. While for patients with a small number of lesions, HyperSHArc may not offer dosimetric benefit compared with conventional IMPT approaches, it could prove advantageous for patients with many small lesions, possibly reducing the risk for brain necrosis. Further optimization of the HyperSHArc planning strategies is foreseen to reduce intermediate dose levels to the brain and increase delivery efficiency. Although the findings of this study need to be verified in larger patient cohorts and, eventually, in preclinical experiments, the demonstrated dose conformity demonstrated in this work provides a proof-of-concept for the potential of HyperSHArc for treating multiple metastases. Further explorative investigation on the potential of HyperSHArc for oligometastasis and multimetastasis cases is planned also for other treatment sites, such as liver metastases.

## Disclosures

The authors declare that they have no known competing financial interests or personal relationships that could have appeared to influence the work reported in this paper.

## Appendix Supplementary materials

HyperSHArc_anonymized_SuppMaterial Image, application 1

## Footnotes

Supplementary material associated with this article can be found in the online version at doi:10.1016/j.adro.2025.101763.
