# Supplementary material for: HyperSHArc: Single-Isocenter Stereotactic Radiosurgery of Multiple Brain Metastases Using Proton, Helium, and Carbon Ion Arc Therapy
Source: Adv Radiat Oncol. 2025 Mar 17;10(5):101763. doi: 10.1016/j.adro.2025.101763 (PMC12013133; doi:10.1016/j.adro.2025.101763)
Supplement: HyperSHArc_anonymized_SuppMaterial [file mmc1.pdf]

Supplementary Material to “HyperSHArc: single-isocenter stereotactic radiosurgery of multiple brain metastases using proton, helium and carbon ion arc”

Lennart Volz[1], Peilin Liu[2], Thomas Tessonnier[3,4,5], Xiaoda Cong[2], Marco Durante[1,6], Andrea Mairani[3,4,5,7,8], Wenbo Gu[9], Amir Abdollahi[3,4,5,7], Xuanfeng Ding[2], Christian Graeff[1,10], Taoran Li[9], Stewart Mein [11,3,5]

[1]*Biophysics, GSI Helmholtz Centre for Heavy Ion Research GmbH, Darmstadt, Germany*

[2]*Department of Radiation Oncology, Corewell Health, Royal Oak, USA*

[3]*Heidelberg Ion Beam Therapy Center (HIT), Heidelberg, Germany*

[4]*Clinical Cooperation Unit Radiation Oncology, German Cancer Research Center (DKFZ), Heidelberg, Germany*

[5]*Heidelberg Institute of Radiation Oncology (HIRO), German Cancer Research Center (DKFZ), Heidelberg, Germany and German Cancer Consortium (DKTK), Heidelberg, Germany*

[6]*Institute of condensed matter physics, TU Darmstadt, Darmstadt, Germany*

[7]*Division of Molecular and Translational Radiation Oncology, National Center for Tumor Diseases (NCT), Heidelberg University Hospital, Heidelberg, Germany*

[8]*National Centre of Oncological Hadrontherapy (CNAO), Medical Physics, Pavia, Italy*

[9]*Department of Radiation Oncology, University of Pennsylvania, Philadelphia, PA, USA*

[10]*Department of electrical engineering and information technology, TU Darmstadt, Darmstadt, Germany*

[11]*Department of Accelerator and Medical Physics, Institute for Quantum Medical Science, National Institutes for Quantum Science and Technology (QST), Chiba, Japan*

S.1 LETd focusing with HyperSHArc-C

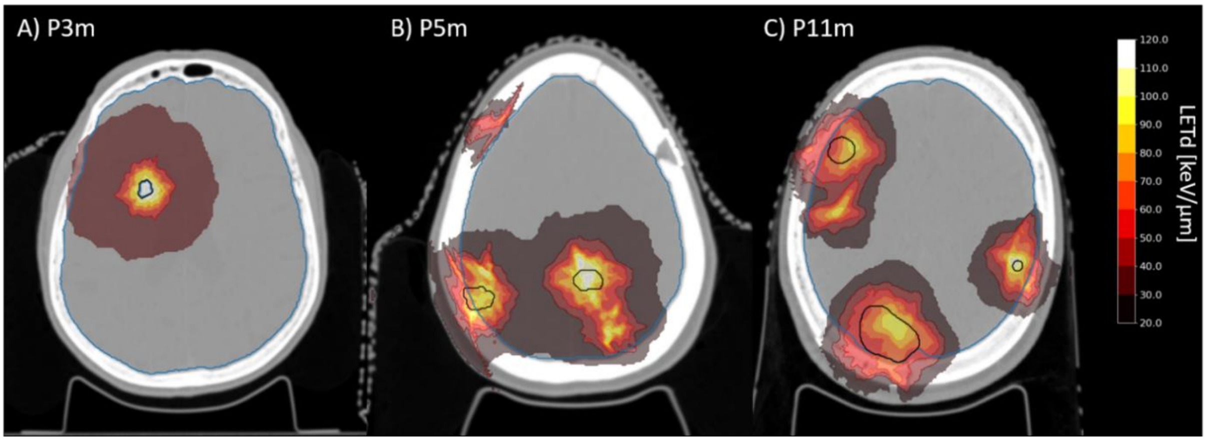

Figure S.1 *Example axial slice of the dose-averaged linear energy transfer (LET$_d$) for P3m (A), P5m (B) and P11m (C) for SHArc(12C). A high LET$_d$ focus is reached within the lesion targets, while the healthy brain is largely spared. The additional high LET$_d$ spots for P5m and P11m belong to an additional out-of-plane target.*

As has also been pointed out in previous literature[14], HyperSHArc-C plans increased the dose averaged linear energy transfer (LETd) in the targets to levels relevant to overcoming tumor radioresistance. While the focus of the main part of the work was on the dosimetric quality of the plans, we also evaluated the LETd focusing achievable with HyperSHArc-C for the multi-

metastases cases. Figure S.1 shows example slices of the LETd distribution overlaid over the CTs for the patients. As expected from the small target size, and energy selection scheme, the LETd was greatly increased inside the GTVs reaching more than 100 keV/µm. Average maximum LETd in the GTVs were 150keV/µm for patient P3m, 135keV/µm for patient P5m, and 121keV/µm for patient P11m. This aspect of HyperSHArc-C may be exploitable for greater therapy effect.

S.2 HyperSHArc-He

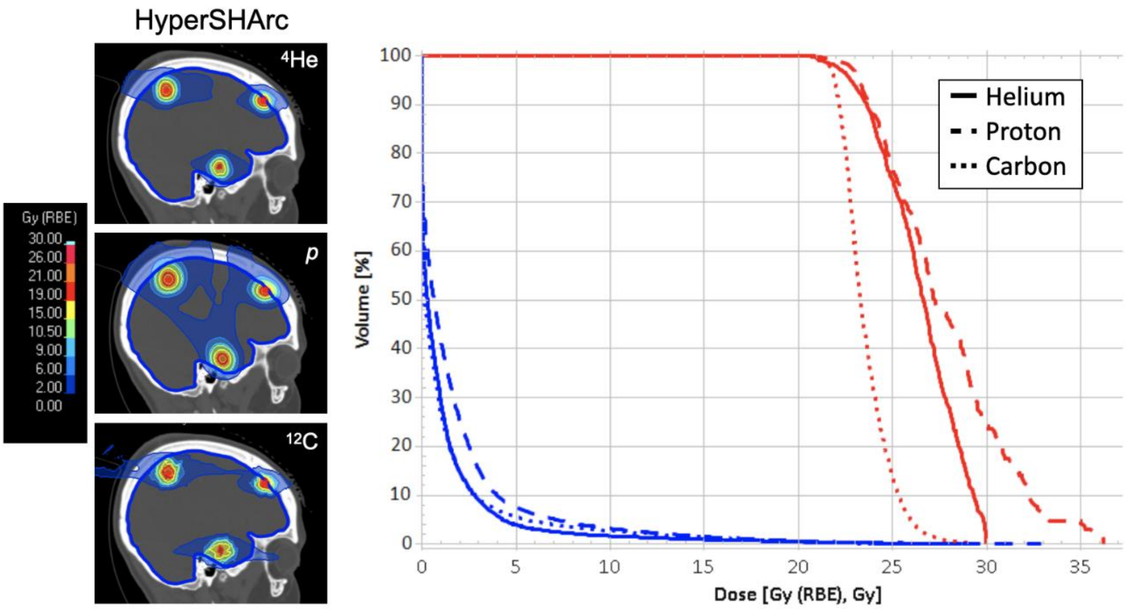

Figure S.2*.Left: Dose maps comparing HyperSHArc using helium against proton and carbon ions for the P5m, applying a 2Gy low-dose threshold. Right: DVH for proton, helium and carbon ions*.

As helium ions have been pointed out as an interesting midpoint between protons and carbon ions, regarding dosimetry, LETd, and delivery complexity, we have optimized a HyperSHArc-He plan P5m. The plan was generated in RayStation[50] employing the mMKM for estimating the RBE, which is also the model of choice for the clinical helium ion therapy program at Heidelberg Ion Beam Therapy Centre[37]. The resulting dose map and DVH is shown in Figure S.2 in comparison to the HyperSHArc-p and HyperSHArc-C results presented in the main article. HyperSHArc-He resulted in a similar dose to the healthy brain compared to HyperSHArc-C, and improved target homogeneity compared to HyperSHArc-p.

S.3 HyperSHArc-C compared to carbon IMPT plans

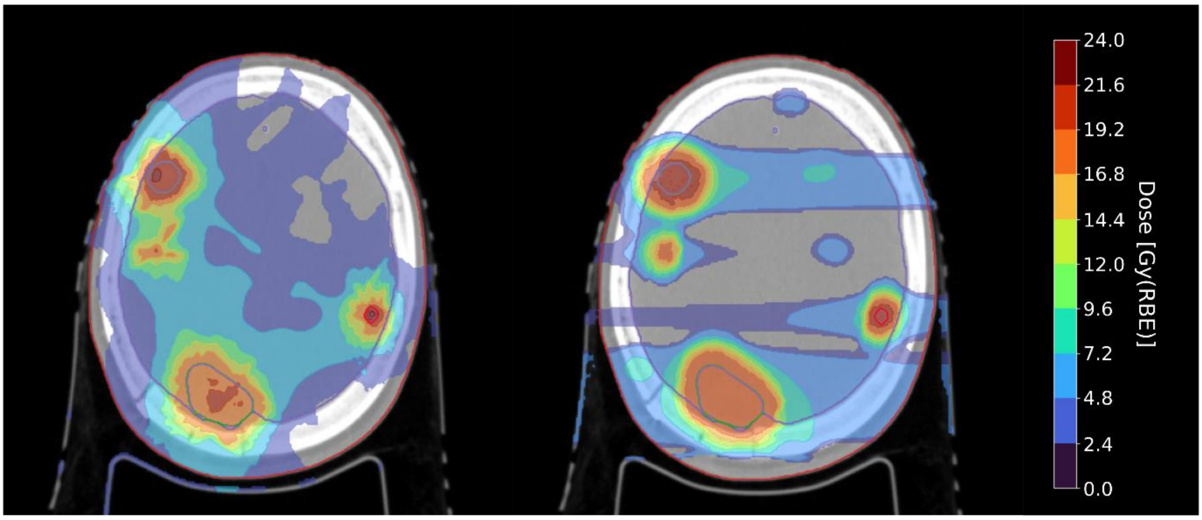

Figure S.3*.Dose maps comparing HyperSHArc using carbon (left) against carbon ion IMPT (right) for P11m, applying a 2Gy low-dose threshold.*

Figure S3 shows a comparison of an example dose slice of the HyperSHArc using carbon ions and three field carbon ion IMPT plan. The dosimetry revealed that V_{12Gy} was reduced by 12 cc for the HyperSHArc-C plan compared to the carbon ion IMPT plans with three fields. The steeper dose gradients, came with an increase in the low dose bath for HyperSHArc-C compared to carbon ion IMPT.
